# Fabrication and Characterization of High-Quality UV Photodetectors Based ZnO Nanorods Using Traditional and Modified Chemical Bath Deposition Methods

**DOI:** 10.3390/nano11030677

**Published:** 2021-03-09

**Authors:** Ahmed Fattah Abdulrahman, Sabah Mohammed Ahmed, Azeez Abdullah Barzinjy, Samir Mustafa Hamad, Naser Mahmoud Ahmed, Munirah Abullah Almessiere

**Affiliations:** 1 Department of Physics, Faculty of Science, University of Zakho, Zakho 42002, Kurdistan Region, Iraq; ahmed.abdulrahman@uoz.edu.krd; 2 Department of Physics, College of Science, University of Duhok, Duhok 42001, Kurdistan Region, Iraq; sabma62@uod.ac; 3 Department of Physics, College of Education, Salahaddin University-Erbil, Erbil 44001, Kurdistan Region, Iraq; 4 Physics Education Department, Faculty of Education, Tishk International University, Erbil 44001, Kurdistan Region, Iraq; 5 Scientific Research Centre, Soran University, Soran 44008, Kurdistan Region, Iraq; samir.hamad@soran.edu.iq; 6 School of Physics, Universiti Sains Malaysia, Penang 11800, Malaysia; naser@usm.my; 7 Department of Biophysics, Institute for Research and Medical Consultations (IRMC), Imam Abdulrahman Bin Faisal University, P.O. Box 1982, Dammam 31441, Saudi Arabia; malmessiere@iau.edu.sa

**Keywords:** UV photodetectors, ZnO nanorods, M-CBD, growth duration, RF sputtering

## Abstract

Ultraviolet (UV) photodetectors (PDs) based on high-quality well-aligned ZnO nanorods (NRs) were fabricated using both modified and conventional chemical bath deposition (CBD) methods. The modified chemical bath deposition (M-CBD) method was made by adding air bubbles to the growth solution during the CBD process. The viability and effectiveness of M-CBD were examined by developing UV PDs based on ZnO NRs. The ZnO nano-seed layer was coated on a glass substrate utilizing radiofrequency (RF) sputtering. The impact of the different growth-times on morphology, growth rate, crystal structure, and optical and chemical properties were investigated systematically using different characterization techniques, such as field-emission scanning electron microscopy (FE-SEM), X-ray diffraction (XRD) analysis, UV–VIS double beam spectrometer, and energy dispersive X-ray analysis (EDX), respectively. The Al/ZnO UV PDs based on ZnO nanorods were fabricated with optimum growth conditions through the two methods of preparation. This study showed that the synthesized ZnO NRs using the M-CBD method for different growth times possess better properties than the conventional method under similar deposition conditions. Despite having the highest aspect ratio and growth rate of ZnO NRs, which were found at 4 h growth duration for both methods, the aspect ratio of ZnO NRs using the M-CBD technique was comparatively higher than the conventional CBD method. Besides, the UV PDs fabricated by the M-CBD method at 5 V bias voltage showed high sensitivity, short response time, quick recovery time, high gain, low dark current, and high photocurrent compared with the UV PD device fabricated by the conventional CBD method.

## 1. Introduction

The ultraviolet (UV) photodetector is a very significant optical device that can be used in a wide area of applications such as biological analysis, environmental analysis, space, optical communication, military, and civilian requests [1]. Currently, different UV photodetectors based on ZnO nanostructures possess considerable concern owing to their exceptional characteristics, such as the direct optical energy band-gap of 3.37 eV, radiation hardness, outstanding sensitivity to absorb oxygen on the surfaces, high optical gain, high chemical stability, and temperature resistance [2]. In these fields of application, rapid response (rise) and retrieval (fall) times are the key UV detection properties that facilitate faster feedback. Up to now, different UV photodetectors through different ZnO nanostructures have been produced utilizing physical and chemical methods on different substrates such as glass, silicon, porous silicon, quartz, sapphire, and flexible substrates (Polyester tape (PET) and Kapton tape) [3]. There are several techniques that are used for synthesizing ZnO materials, such as chemical bath deposition (CBD) method, spray pyrolysis, hydrothermal synthesis, chemical vapor deposition, sol-gel technique, vapor phase transport, successive ionic layer adsorption reaction (SILAR), electrodeposition, and modified chemical bath deposition (M-CBD) [4]. Among all these growth methods mentioned above, the M-CBD method is a fascinating technique that possesses high performance, and it is the most effective and efficient method for UV photodetector fabrication based on different ZnO nanostructures. Additionally, The M-CBD method is also well recommended for its simplicity, low cost, low temperature (<100 °C), high-quality crystal and large capacity of growth vessel used, reproducibility, and non-hazardousness. Moreover, M-CBD method does not require a complex growth system and conductive substrates, and starting chemicals are commonly cheap and available [5,6]. Similarly, the M-CBD method solves or addresses all limitations or problems of CBD methods that were mentioned from previous works [7]. The modified CBD process is introduced by involving air bubbles in the growth solution through the CBD apparatus in the oven, which unceasingly carries the continuous precursor meditation of the deposition solution to the substrates. Consequently, it decreases the probability of standardized response and provides the oxygen throughout the CBD reaction [5].

In M-CBD, different deposition parameters can directly affect the ZnO structures and quality such as shape, size, and defects which have significant impact on the performance of UV photodetectors. These deposition parameters are precursor concentration, temperature, growth duration, pH value, type of substrate, and seed layer geometry [8]. The growth duration in the M-CBD method is one of the significant deposition conditions or parameters that can directly govern the shape, size, structure quality, and density distribution of ZnO nanostructures, which in turn directly influences the efficiency of UV photodetectors [9]. In this study, the Al/ZnO UV photodetectors based on vertically aligned ZnO nanorods (NRs) were fabricated on glass substrates by means of modified and conventional CBD methods. Similarly, the effects of the growth duration of the ZnO nanorod properties grown by the two ways of preparations were systemically investigated. Additionally, the photodetection characterizations based on the optimum growth duration for ZnO nanorods by the two ways of preparation were obtained. The novelty of this study can be highlighted, especially, when air bubbles are involved during the growth process, as the ZnO NRs became more uniform and homogeneously distributed over the seeded substrate. Properties such as average diameter, average length, aspect ratio, and growth rate were dramatically improved. The energy band gap and the ratio between Zn and O was almost 1:1 for all the analyzed samples, which shows that adding air bubbles does not change this stoichiometric ratio. Interestingly, adding air bubbles caused the formation of single crystal due to the regular alignment of the ZnO NRs and an accordingly decreasing full width at half maximum (FWHM), which led to producing high quality ZnO NRs. Additionally, the volume of hexagonal cell, bond length, spectral responsivity, magnified (on/off) Photoresponse cycle, and absorptivity of ZnO Nanorods increased with the M-CBD.

## 2. Materials and Methods

In this study, deionized (DI) water was used for growth and treatment processes. Additionally, chemicals without further purification were used in this work, such as Hexamethylenetetramine (HMTA) (C_6_H_12_N_4_) and Zinc Nitrate Hexahydrate (ZNH) (Zn(NO_3_)_2_·6H_2_O), purchased from Sigma-Aldrich Company (Baghdad, Iraq).

### 2.1. ZnO Seed Layer Preparations

ZnO nano-seed layers were deposited on glass substrates through three steps. The first step was as follows: a microscopic glass substrate was cleaned in an ultrasonic bath via ethanol, acetone, and deionized water for 15 min, correspondingly, and then dried up with nitrogen gas. The next step was as follows: 100 nm of the ZnO seed layer was coated on the prepared glass substrates through radiofrequency (RF) sputtering by means of ZnO target (99.99% purity of ZnO) with 5.5 × 10^−3^ mbar gas-pressure of argon in the RF chamber and 150 Watt radiofrequency power for 15 min. The last step was as follows: the seeded substrates were annealed inside the tubular furnace under an atmosphere at 400 °C for 2 h [10].

### 2.2. ZnO Nanorods Growth

The modified and traditional CBD methods were utilized to synthesize vertically aligned ZnO NRs. The precursors that were employed in this work were (C_6_H_12_N_4_) (HMTA) and (Zn(NO_3_)_2_·6H_2_O) (ZNH); also, DI water was used as a solvent. A suitable amount of both HMTA and ZNH was dissolved separately in DI water, and two dissolved solutions were well mixed using a magnetic stirrer to get the homogenous growth solution [10,11]. The prepared growth solution was transparent, and the initial pH value of the growth solution was about 6.7. To investigate the effects of the M-CBD method on the ZnO properties compared with the conventional CBD method, the ZnO NRs were prepared by two methods for different growth duration. The growth times were 0.5, 1, 2, 3, 4, and 5 h and the corresponding samples when air bubbles did not exist were labeled a, b, c, d, e, and f, respectively. In the cases that air bubbles did exist, the corresponding samples were labeled g, h, i, j, k, and l. The initial and final pH values of the growth solution were measured precisely before and after the growth process. The seeded substrates were introduced inside a beaker at 70 degrees containing a combination of the two growth solutions. Then the CBD arrangement (beaker with seeded substrate and solution) was placed inside the oven under air bubbling with 5000 mLit./min flow rate and 70,000 Pascal air pressure at 95 °C for different growth durations [5,11]. After completing the required time of the growth process, the prepared ZnO NR samples were washed using DI water to take off the remaining salts, and nitrogen gas was utilized to dry it.

### 2.3. Fabrication of UV Photodetectors

**Figure 1:**
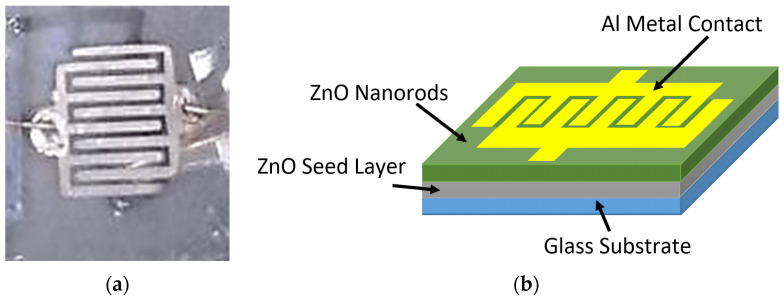
(**a**) Graphical image and (**b**) schematic illustration for the fabricated UV photodetectors (PDs).

After the growth of ZnO NRs on a glass substrate via modified and conventional CBD methods, the 120 nm thickness of Aluminum (Al) grid target with (99.99%) purity was deposited on the top of ZnO NRs by means of a metal mask to fabricate the UV photodetectors based on ZnO nanorods. RF sputtering was used to deposit the electrodes. The deposition cavity was discharged at 3 × 10^−3^ mbar with an RF power of 120 W for 30 min. The construction of the contact metal Al grid comprised two interdigitate contacts (electrodes) with five fingers. The width and length of each finger were 0.035 cm and 0.54 cm, respectively, and the distance spacing between the fingers was 0.04 cm. The ZnO nanorods’ UV detector had an active area of about 0.171 cm^2^. The structure and the photographical image of the fabricated UV photodetectors are shown in Figure 1. ZnO UV photodetectors were fabricated based on optimum growth condition, and the duration was 4 h for the two ways of preparation.

### 2.4. Characterization Techniques

**Figure 2:**
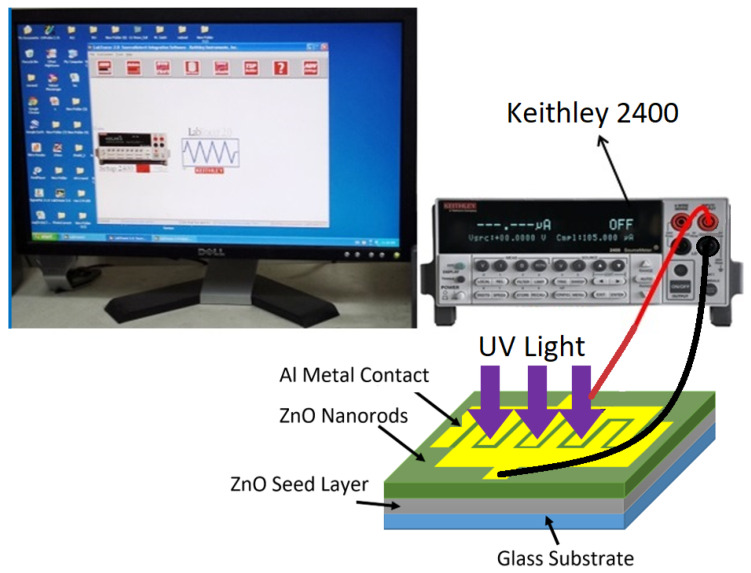
Electrical system for electrical current-voltage (I-V) characteristics measurement of UV photodetectors.

The radiofrequency (RF) sputtering magnetron system Auto HHV 500 sputter coater model Auto 500 was utilized to deposit the ZnO seed layer onto the substrates and contact the metal Al grid for UV PDs. The annealing tube furnace model Lenton VTF/12/60/700 was utilized to anneal the ZnO seed layer. The high-quality pH meter benchtop model PHSJ-4F from Hinotek Company (Ningbo, China) was employed to measure the pH of the deposition solution. Moreover, the three standard buffer pH solutions (pH 4, pH 7, and pH 10) were utilized to adjust the pH meter. Furthermore, a field-emission scanning electron microscope (FE-SEM) (model: FEI Nova a nano SEM 450 Netherlands and Leo-Supra 50 VP, Carl Zeiss, Oberkochen, Germany) was utilized to characterize the surface morphology of the samples, and energy dispersive X-ray spectroscopy (EDX) was used to measure (quantitative and qualitative) the elemental composition of ZnO nanorods. The crystal structure of ZnO nanorods was examined using high-resolution XRD (Rigaku, Stuttgart, Germany; X-Pert Pro MRD model with CuKa (λ = 0.154050 nm) and a scanning range of 2θ set between 20° and 80°). Additionally, a double beam UV-visible (UV-4100) spectrometer with a wavelength range of 300 to 800 nm was utilized to illustrate the optical characteristics and energy band gap of ZnO samples that were calculated from the transmittance spectrums of ZnO NRs. The fabricated UV photodetectors based on ZnO NRs were characterized using current voltage (I-V) characteristics quantities. The measurements were steered in dark and under the UV light of 380 nm through an intensity of 0.61 mW/cm^2^. The electrical characteristics of the UV photodetectors, including sensitivity, response time, recovery time, gain, and other parameters, were measured using a Keithely 2400 high-voltage source unit connected to a PC for data analysis, as shown in Figure 2.

## 3. Results and Discussion

### 3.1. Morphology Characteristics

**Figure 3:**
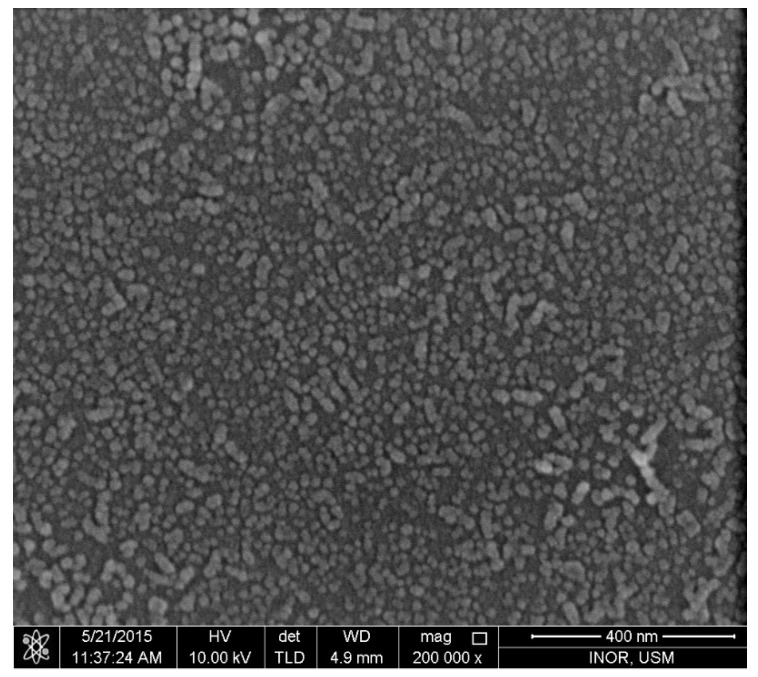
The ZnO nano-seed layer deposited on glass substrates before fabricating ZnO nanorods (NRs) on it.

Figure 3 shows the ZnO nano-seed layer which was deposited on glass substrates before fabricating ZnO NRs on it, as described previously.

**Figure 4:**
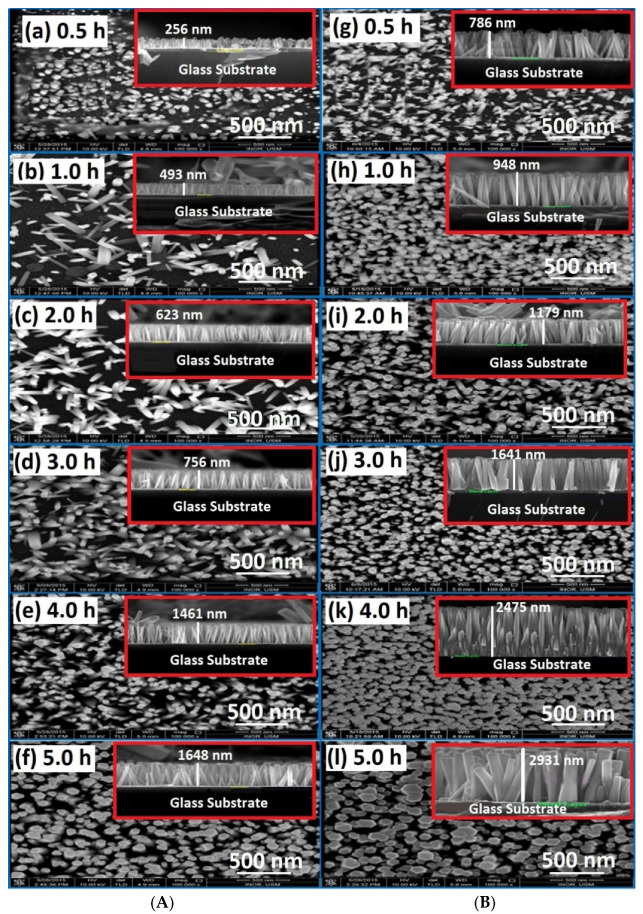
FE-SEM images of ZnO nanorods grownup for different growth periods: (**A**) no air bubbles and (**B**) air bubbles. The inset at the top-right corner represents the cross-section of ZnO nanorods for each growth time representing the cross-section of nanorods. ((**a**,**g**) for 0.5 h, (**b**,**h**) for 1.0 h, (**c**,**i**) for 2.0 h, (**d**,**j**) for 3.0 h, (**e**,**k**) for 4.0 h, (**f**,**l**) for 5.0 h growth time respectively).

The FE-SEM images of ZnO NRs fabricated on glass substrates by both conventional (without air bubbles) and modified (with air bubbles) methods at different growth durations are shown in Figure 4. From the surface morphology of ZnO NRs (Figure 4a–l), it can be clearly seen that at a low growth time of 0.5 h, as shown in Figure 4a, the non-uniform distribution, short length, and low density of ZnO NRs are formed with random orientation over the entire substrates. However, with increasing growth time, a significant change in the surface morphology of ZnO NRs regarding the length and diameter was observed [3]. It is clear, from Figure 4b–f, that the morphology of ZnO NRs was improved by vertical well-alignment, density distribution, and its hexagonal shape, longer length, and bigger size. Once the air bubbles are involved during the growth process, as seen in Figure 4g–l, the fabricated ZnO NRs became more uniformly and homogeneously distributed over the seeded substrate, in comparison with the case where no air bubbles were introduced. This may be related to the fact that the air-bubbles agitated the growth solution inside the CBD reactor and help to overcome the precipitation of heavy precursors at the bottom of the beaker during the preparation processes of ZnO NRs. These attitudes have a significant role in homogenizing the solution and driving factor to reach a uniform temperature, pH, and concentration distribution as well as enhancing the chemical reactions over the substrate. The weighty forerunners were hastened at the bottommost section of the beaker, and this was the most problematic phenomena when the air bubbles were not involved. Accordingly, from a morphology perspective, one can notice that the growth-time has very important consequences on the ZnO structure characteristics. The FE-SEM results were in good agreement with previous works [12,13,14].

**Figure 5:**
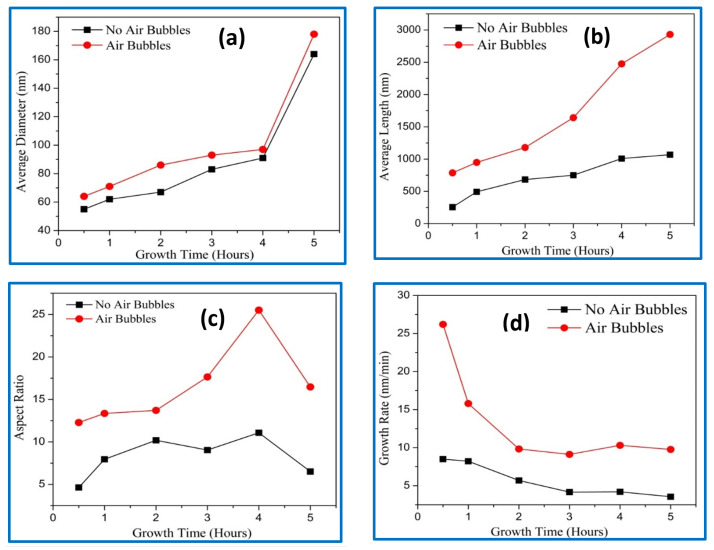
Effect of different growth times on the ZnO NRs grown using conventional and modified chemical bath deposition (CBD) methods: (**a**) average diameter, (**b**) average length, (**c**) aspect ratio, and (**d**) growth rate.

The average diameter, length, aspect ratio (length/diameter), and growth rate of the ZnO NRs with different growth durations are shown in Figure 5a–d. The average diameters of the ZnO NRs were calculated from the top view of the FE-SEM images, and they are presented in Figure 5a. Increasing the diameter of ZnO nanorods with growth times can be correlated to the combination of the adjacent nanorods and forming new ones with larger diameters [15,16]. In addition, more growth time means giving more chances to join more atoms together to form ZnO nanorods with a larger diameter. It can be noticed that, compared with the conventional method, the nanorods, using the modified way, possess larger diameter. Figure 5b shows the average length of ZnO NRs grown by conventional and modified methods. The average length increased with durations for both NRs with air bubbles and those without. However, the average length using a modified method was longer than the average length of ZnO NRs using a conventional way. In the modified CBD method, the growth duration rate should be reduced by 2.5 for growing the same length compared with the conventional CBD method. For instance, to obtain nearly the same length of ZnO NRs on the basis of growth times, 0.5 h are needed using a modified CBD method, while 3 h are needed for the same length using a conventional process. The acquired average length and diameter using the conventional way were in good agreement with the literature [17,18,19]. The alteration in an aspect ratio of ZnO nanorods versus growth times are shown in Figure 5c. It can be seen that the aspect ratio of the ZnO NRs gradually increased with growth time from 0.5 h to 4 h. Further increasing of growth time to 5 h, the aspect ratios rapidly decreased. The sudden change in the aspect ratio was possibly due to differences in average diameter and length of the ZnO NRs. The behavior of aspect ratio without air bubbles was in good agreement with previously reported studies [20,21,22,23]. From Figure 5c, we noticed that the optimum aspect ratio corresponding to the growth time was achieved at 4 h. The obtained aspect ratios of ZnO NRs were (11) and (25) for conventional and modified CBD methods at 4 h, respectively. Moreover, the aspect ratio of ZnO NRs with air bubbles was higher than the aspect ratio of ZnO NRs with no air bubbles at the same growth duration. This is a good indicator, showing that better crystal quality can be obtained using air bubbles in the CBD method. The higher aspect ratio can be regarded as a key factor for determining the energy-conversion efficiency of solar cells [24,25]. The growth rates versus times are revealed in Figure 5d. For both methods, the growth rate was increased with growth times. This shows that the higher growth rate was observed in the modification method, since with air bubbles the ZnO NRs grow longer compared with ZnO nanorods with no air bubbles. It is believed that the air bubbles stimulate the growth process of nanorods laterally (diameter) and vertically (length). The corresponding growth rate behaviors using the conventional way were in good agreement with previous studies [26,27].

### 3.2. The pH Values of the Growth Solutions

**Figure 6:**
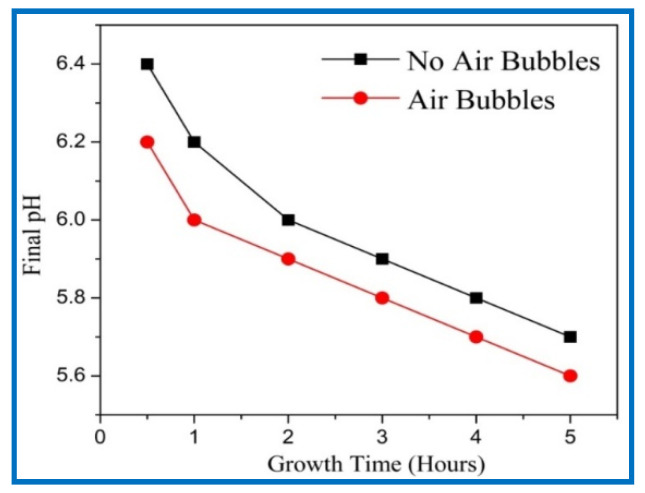
Effect of growth times on final pH value of growth’s solution when ZnO nanorods were fabricated, in the case of no air bubbles and in the case of air bubbles.

The final pH values of growth solution with growth time from 0.5 to 5 h using both methods are shown in Figure 6. The initial value of all samples was 6.7 before starting the reaction. The final pH values were measured very carefully after the growing process. Figure 6 reveals that the final pH values were decreased with growth times, since the OH¯ ions became lower at higher pH value in the solution [28]. The growth of the nanorods can be considered as a competition between the time rate and the dissolution rate present in the growth solution [29]. A higher growth time gives a higher dissolution rate, which is what took place at higher pH values. Additionally, it can be observed that the final pH value for air bubbles was lower than the final pH with no air bubbles in the growth solution. This is possibly related to the agitation of growth solutions and provides homogeneity during the CBD growth. These results support the higher growth rate, aspect ratio, higher density, vertical alignment, hexagonal shape, and homogeneity of ZnO NRs.

### 3.3. Chemical Analysis of the Grown ZnO Nanorods

**Figure 7:**
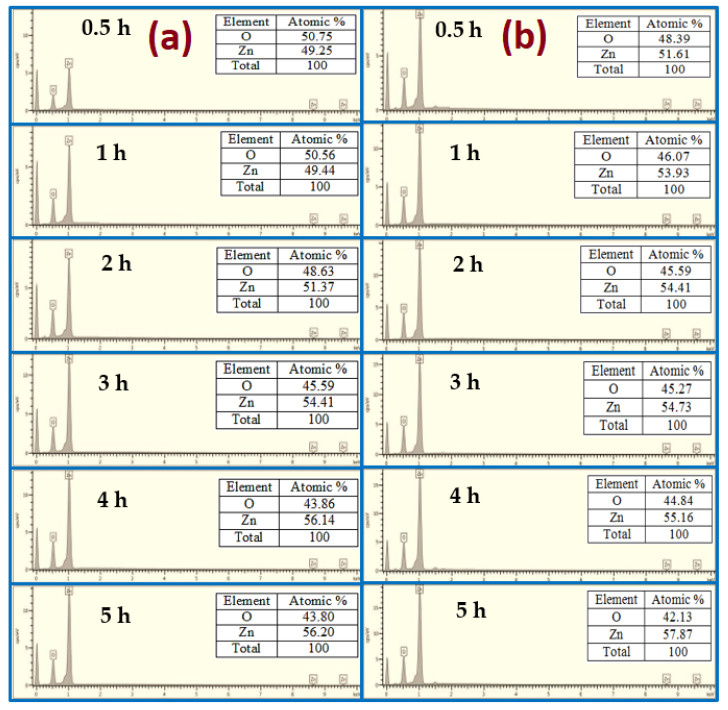
EDX analysis of ZnO NRs for different growth durations: (**a**) no air bubbles and (**b**) air bubbles.

The elemental compositions using EDX analysis of the as-grown ZnO at different growth times using both conventional and modified CBD methods are shown in Figure 7. The EDX analysis displays the presence of Zn and O, which matches to the composition of ZnO, deprived of the presence of any impurities or substrate sign, confirming that the grown samples are pure ZnO. The ratio between Zn and O is almost the same for all analyzed samples. The molecular ratio of Zn/O calculated quantitatively by EDX was almost 1:1. Similar findings have been obtained by Chong et al. [30].

### 3.4. X-ray Diffraction (XRD) Analysis

**Figure 8:**
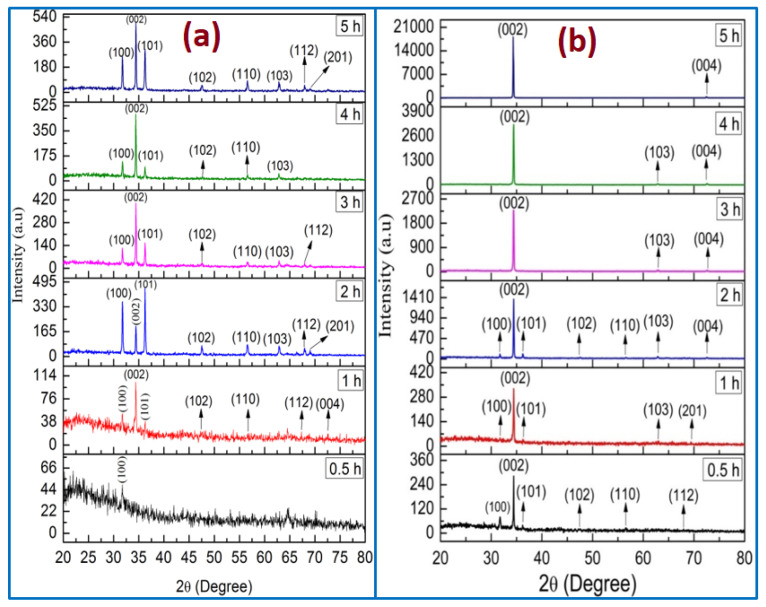
X-ray diffraction patterns of ZnO nanorods grown for different growth durations: (**a**) no air bubbles and (**b**) air bubbles.

The XRD patterns of ZnO (using two methods) of different growth durations are displayed in Figure 8. All the diffraction peaks have been indexed as what the wurtzite hexagonal structure of ZnO nanorods corresponded to (JCPDS cards No. 01-080-0075). In addition, no other diffraction peaks from impurities were detected, confirming that high purity of ZnO nanocrystal was performed, which was also supported by EDX analysis. For all the ZnO samples, the peaks observed at 2θ = 34.4 and 34.3 demonstrated the favorably oriented growth along the c-axis. The ZnO NRs tended to be grown in the (002) plane because the surface free energy of this orientation was the lowest one compared with the others, such as the (100) and (101) planes [31,32].

In the case of no air bubbles, the (002) diffraction peak from the XRD pattern is dominant for the different times of 1, 3, 4, and 5 h, as shown in Figure 8a [11], whereas the XRD pattern for 0.5 h revealed a semi-amorphous characteristic, which is in a good agreement with morphology characteristics. Additionally, the ZnO grown for 2 h is described in Figure 8a. The (101) is dominant with a strong diffraction peak, indicating that the ZnO NRs oriented along (101) axis [33]. In the case of no air bubbles, the obtained XRD consequences were in a respectable agreement with the FE-SEM morphology and the preceding works [34,35]. The diffractions peaks from another surface, such as (100), (101), (102), (110), (103), and (112), point to a significant enough number of ZnO NRs to be oriented in these directions [34]. Conversely, when air bubbles were involved in the growth solution, the ZnO NRs’ (002) diffraction peak in all XRD patterns was dominant with growth time from 0.5 to 5 h as displayed in Figure 8b. The narrow, sharp, and strong ZnO (002) peaks in the XRD patterns recognized that the ZnO NRs were synthesized along the c-axis with wurtzite hexagonal structure [36,37]. From all the analysis results of ZnO NRs, it can be observed that the air bubbles and growth times played a vital role in the growth of the ZnO structure.

**Figure 9:**
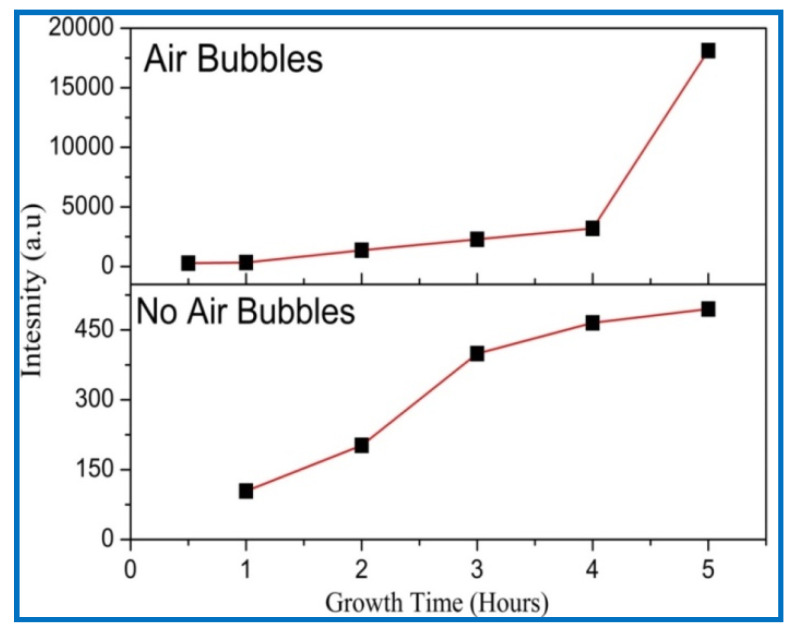
Growth time versus intensity of ZnO NRs of diffraction peak (002) plane when no air bubbles are present and when air bubbles are present.

The (002) peak intensity of ZnO NRs with growth time is shown in Figure 9. It can be noted that the diffraction peak’s (002) intensity becomes higher and narrower as growth times increases, demonstrating that the crystal quality of ZnO is enhanced with growth time. Additionally, when air bubbles are involved, the intensity of the ZnO NR’s (002) peak along the c-axis is higher than the intensity of the (002) peak without air bubbles. This signifies that the ZnO crystal quality becomes better when air bubbles are involved. The increasing peak intensity (002) with growth time is in good agreement with previous studies [38,39].

The structural properties of ZnO nanorods, such as peak position (θ), intensity, lattice constants (*a* and *c*), and the internal strains (Ƹ*_c_* and Ƹ*_a_*) along diffractions peak (002) for both methods, are recorded in Table 1 and Table 2 independently. The lattice constants (*a* and *c*) of the ZnO were obtained using Bragg’s law [40].

(1) $$a=\;\sqrt{\frac{1}{3\;}}\frac{\lambda }{\sin\theta }$$

(2) $$c=\frac{\lambda }{\sin\theta }$$

where λ is the wavelength of the X-ray source and θ is the angle of the diffraction peaks.

The strains (Ƹ*_c_*) and (Ƹ*_a_*) of the ZnO along c-axis and a-axis, correspondingly, are calculated from the following equations [40]:

(3) $$\varepsilon_{c}=\frac{c-c_{o}}{c_{o}}\times 100\%$$

(4) $$\varepsilon_{a}=\frac{a-a_{o}}{a_{o}}\times 100\%$$

where *a_o_* and *c_o_* represented the standard lattice constants for unstrained ZnO structures that existed in the database.

In the absence of air bubbles, the strains (Ƹ*_c_*) and (Ƹ*_a_*) had the same trend; they were decreased with growth duration up to 4 h and then increased with extra growth period to 5 h, as listed in Table 1.

These variations in strain are due to changes in the lattice parameters (*a*, *c*, and *d*) as a result of being a disparity among ZnO NRs and glass substrate. The strain consequences were in respectable agreement with the literature [22].

The lowest compressive strain along the (002) diffraction peak from ZnO NRs was obtained for 4 h of growth time. This suggests a more ordered ZnO crystal quality grown on the seeded substrate. In the case involving air bubbles, the strains (Ƹ*_c_*) and (Ƹ*_a_*) are listed in Table 2. In this case, the strains (Ƹ*_c_*) and (Ƹ*_a_*) were decreased as the growth time increased from 0.5 to 5 h. In both methods, the negative values of strain imply the compressive strain within all the samples.

The interplanar distance of ZnO from (002) peak was found according to Bragg’s law, and its results are summarized in Table 1 and Table 2 for the two preparation methods [40].

(5) $$\frac{1}{d^{2}}=\frac{4}{3}\left(\frac{h^{2}+\mathrm{hk}+k^{2}}{a^{2}}\right)+\frac{l^{2}}{c^{2}}$$

where a and c are the lattice coefficients.

**Figure 10:**
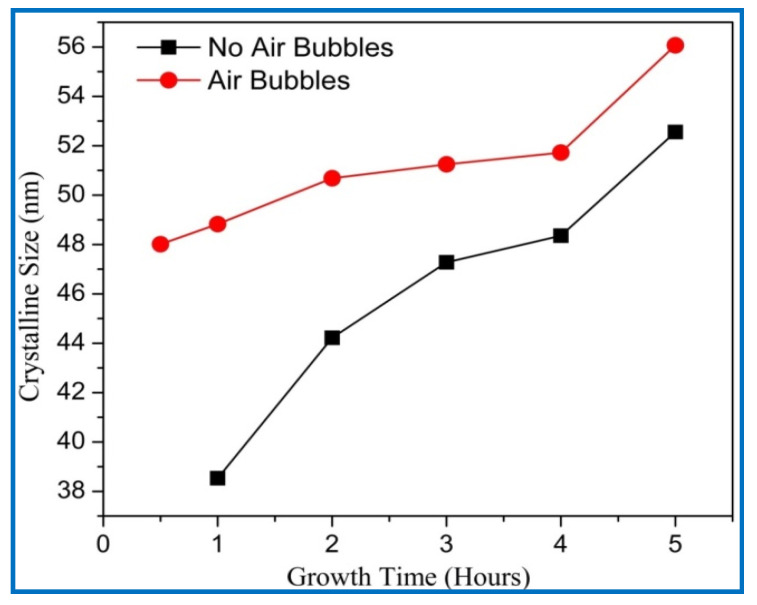
Variation of crystalline size of ZnO nanorods of diffraction peak (002) with growth time for both cases: air bubbles not involved and air bubbles involved.

Likewise, the average crystalline size of ZnO along (002) diffraction peaks as revealed in Figure 10 are calculated from the Debye–Scherer formula [40].

(6) $$D=\frac{k\lambda }{\beta \cos\theta }$$

where k is constant, which is taken to be 0.9; λ is the wavelength of the X-ray source; β is full width at half maximum (FWHM) in radian; and θ is the Bragg diffraction angle.

**Figure 11:**
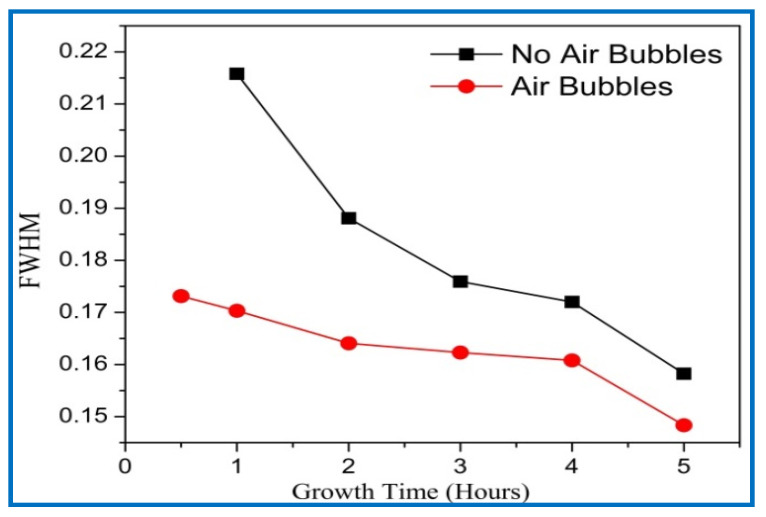
Full width at half maximum (FWHM) of ZnO nanorods of diffraction peak (002) with growth time for both cases: air bubbles not involved and air bubbles involved.

The average crystalline sizes of ZnO NRs prepared by both methods had similar behavior, and they were increased with increasing growth time. From Figure 10, the average crystal size of ZnO NRs grown by modification method displays a larger size than the one from the conventional method. According to the Dedye–Scherrer formula, the reduction of FWHM values with increasing growth times resulted in the increase of crystalline size and improvement in the crystal quality (Figure 11). With increasing the growth durations, more hexamethylenetetramine (HMTA) could have decomposed to OH¯ inducing further grain growth [41]. Consequently, more growth time gives more chance to rejoin extra adatoms of Zn^+^ and OH^−^ ions together to the final product, and then increases the crystalline size of ZnO [42]. The average crystalline size of ZnO NRs by the conventional way was in a good agreement with the previous literature [38,39,43].

**Figure 12:**
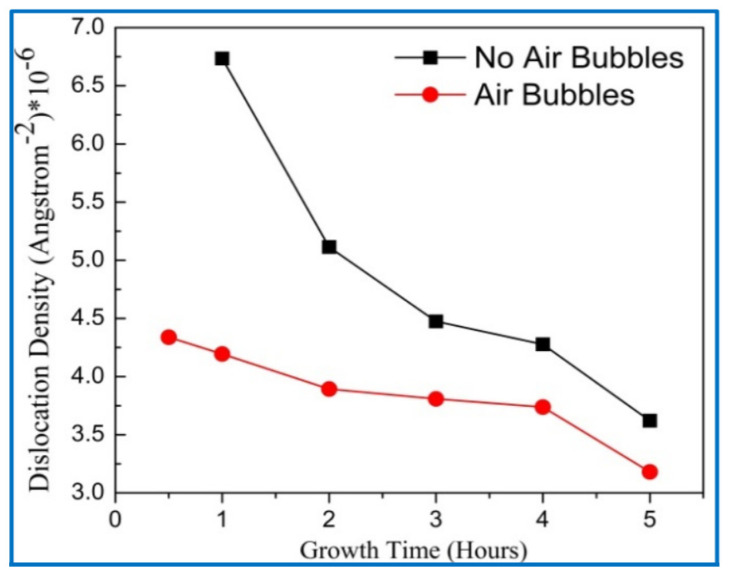
Variation of dislocation density with growth times for both preparation methods (conventional and modified) respectively.

The dislocation density (δ) along with the diffraction peak (002) represents the number of defects in the crystal as shown in Figure 12, and it is calculated from the following equation [44]:

(7) $$\delta =\frac{1}{D^{2}}$$

where D is the crystalline size.

From Figure 12, it can be seen that the dislocation densities for the two methods gradually decreased with growth times up to 5 h; this means that the defects in the crystal are decreased with growth times [45]. This, perhaps, related to increasing the crystalline size, since the dislocation density is inversely proportional to the square of crystal size as well as enhancement of the lattice structure of ZnO NRs. Additionally, the dislocation density for no air bubbles was higher than the dislocation density when air bubbles are involved. This can validate that the crystal quality of ZnO NRs is improved with air bubbles more than the quality of ZnO with no air bubbles.

**Figure 13:**
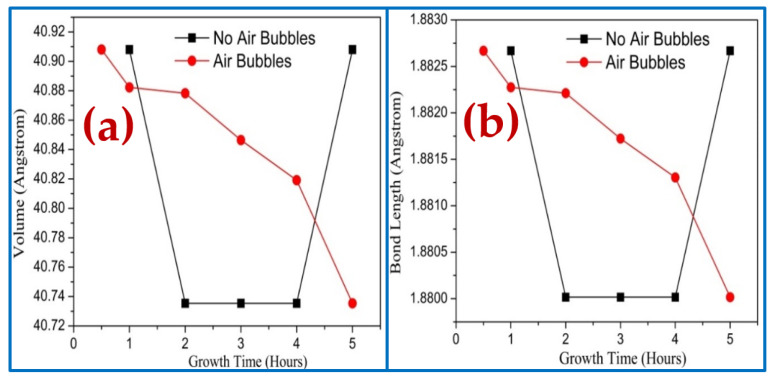
Effect of growth times on ZnO NRs of diffraction peak (002) fabricated where air bubbles were not involved and where air bubbles were involved: (**a**) volume of hexagonal cell and (**b**) bond length.

The impact of the dissimilar growth times on the volume of hexagonal cell and bond length is shown in Figure 13a,b, respectively. The bond length and volume are calculated from the following equation [38]:

(8) $$L=\sqrt{\frac{a^{2}}{3}+{\left(\frac{1}{2}-u\right)}^{2}c^{2}}$$

where u is the positional parameter in the wurtzite structure and is related to c/a ratio; u is a measurement of displaced atoms concerning the next along the c-axis and is given by the following equation [38]:

(9) $$u=\frac{a^{2}}{3c^{2}}+0.25$$

The volume (V) of the hexagonal cell can be evaluated by the following equation [38]:

(10) $$V=\frac{\sqrt{3}}{2}a^{2}c$$

where a and c are the lattice constants.

These results show that both bond length and volume cells follow the same behavior. In the absence of the air bubbles, the volume and bond length are sharply decreased along with the growth time from 1 to 2 h, whereas they stayed constant from 2 to 4 h and then rapidly increased with growing time up to 5 h. However, the volume and bond length decreased dramatically with growth time from 0.5 to 5 h when air bubbles were involved. This variation of volume and bond length along diffraction peak (002) was due to the improvement of the lattice parameters with growth duration as a result of increasing grain size, reducing defects, and dislocation density, which followed that improvement of crystal quality [46].

### 3.5. Optical Properties of ZnO Nanorods

**Figure 14:**
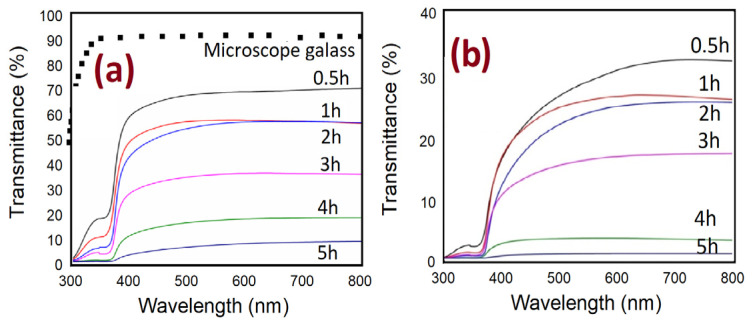
Optical transmittance spectrums of ZnO nanorods fabricated at different growth durations for both methods of preparation: (**a**) conventional CBD and (**b**) modified CBD.

**Figure 15:**
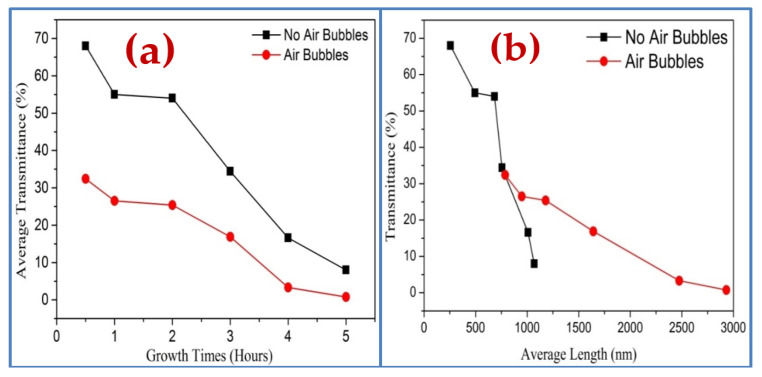
Optical transmittance spectrums of ZnO nanorods fabricated by two ways of preparation versus (**a**) different growth times and (**b**) average length of ZnO nanorods.

The UV-visible spectrometer is utilized to study the impact of the different growth times on the optical characteristics of ZnO nanorods grown on substrates by both methods. The optical transmittance spectrum from 300 to 800 nm of ZnO nanorods is shown in Figure 14. It can be noticed, from Figure 14a,b, that the spectrums possess high transmittance in the visible region and low transmittance in the UV region for all ZnO samples. In general, the spectrum shows the decrease of transmittance with increasing growth durations. This is possibly related to enhancing the scattering effect in ZnO NRs grown at a longer time in two ways that have thicker ZnO NRs [47,48,49]. Additionally, the transmittance spectrum, in the absence of air bubbles, is almost two times higher than the transmittance spectrum when air bubbles are involved (Figure 15). The reason behind that is, perhaps, related to the thickness, the length of NRs, growth time, and density distribution of ZnO nanorods, as is illustrated in Figure 15a,b. The overall results show the light absorption reducing exponentially with increasing thickness.

Moreover, it can be seen, in Figure 15, that with increasing growth time, the transmission spectra from ZnO are shifted to the higher wavelength. This is referred to as the reduction of energy band gap [50], which possibly originated from the interior stress formed in the film and the light scattering properties by the random distribution of the nanorod’s orientations [51]. The transmittance spectrum decreases sharply around 390 nm at 4 h of growth time with air bubbles, and it is around 385 without air bubble; this shift is most likely due to differences in the thickness, defects, and surface roughness of the samples prepared by both ways [52].

**Figure 16:**
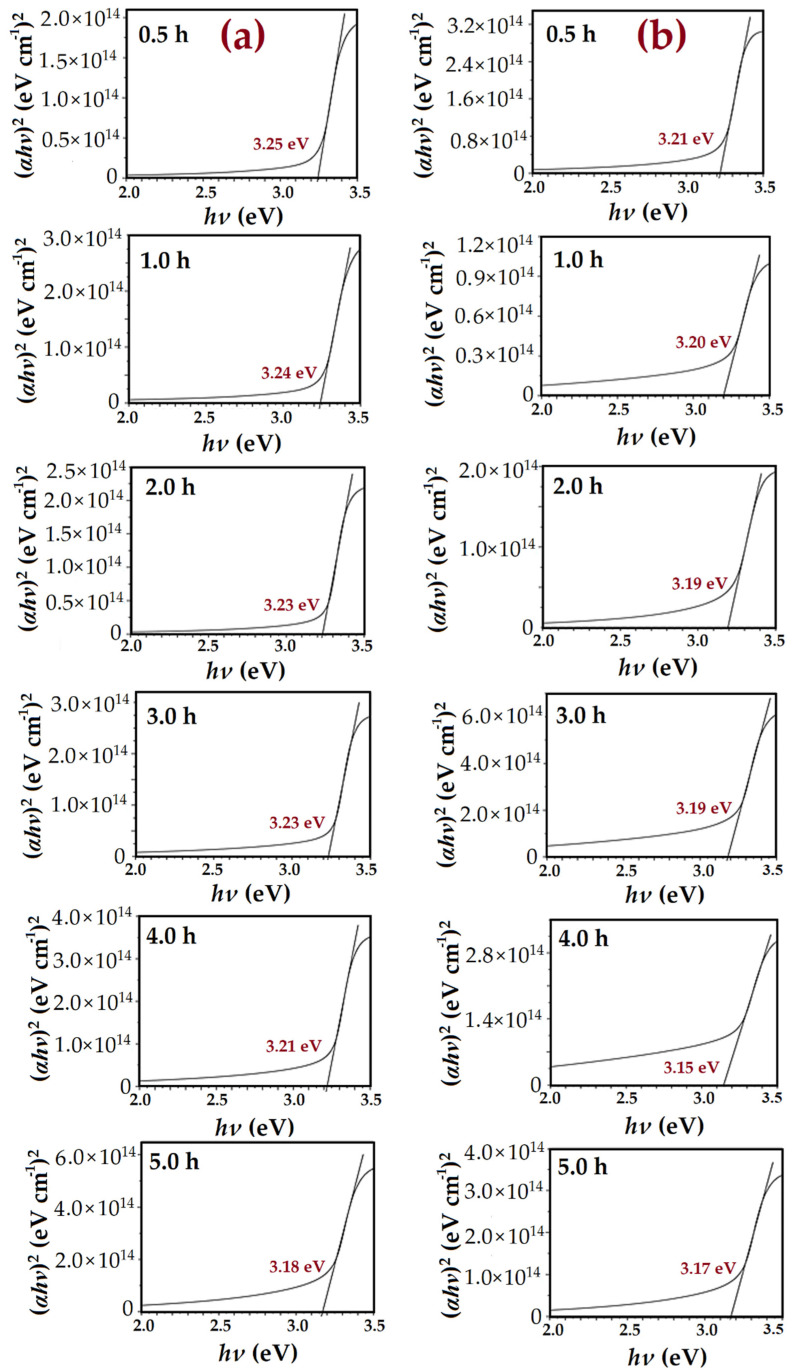
Variation of (*αhν*)^2^ against applied energy band gap (*hν*) of the ZnO nanorods synthesized at diverse growth times: (**a**) conventional CBD method and (**b**) modified CBD method.

The extrapolation of (*αhν*)^2^ versus *hν* plots was derived from the transmittance spectrums [53], and then used to calculate the energy band gap (E_g_) of ZnO samples, as shown in Figure 16.

(11) $${\left(\alpha h\nu \right)}^{2}=A{\left(h\nu -E_{g}\right)}^{n}$$

where *α* is the absorption factor, *hν* is the photon energy, *A* is constant, E_g_ is the optical band-gap energy, and *n* relies upon the transmission type (equals to 1/2 for permitted straight transmission). For the transmittance spectrum, the (*α*) coefficient can be calculated by the following equation: [54]

(12) $$\alpha =\frac{\ln\left(\frac{1}{T}\right)}{d}$$

where T is the transmittance of the ZnO films and d is the film thickness.

Figure 16a,b shows that the transition region is about 3.2 eV and 3.15 eV for ZnO NRs without and with air bubbles, correspondingly. This corresponds to the direct transition band gap energy E_g_ of ZnO semiconductor [55]. Normally, the E_g_ is concerned with the stress state, carrier concentration, and grain sizes in the material [56]. The estimated E_g_ by the conventional method was 3.250 eV, 3.240 eV, 3.2334 eV, 3.230 eV, 3.221 eV, and 3.170 eV at the different growth times 0.5, 1, 2, 3, 4, and 5 h, correspondingly. Meanwhile, E_g_ by the modified method was 3.22 eV, 3.20 eV, 3.19 eV, 3.18 eV, 3.15 eV, and 3.16 eV for the diverse growth times from 0.5 to 5 h, respectively. It can be seen that the energy band gap of ZnO with the existence of the air bubbles was lower than the E_g_ with no air bubbles. Accordingly, this has an obvious impact on enhancing the crystallite size and crystallinity of ZnO NRs, particularly on the samples that were grown with the existence of the air bubbles. The maximum of the E_g_ of ZnO NRs grown at 0.5 h in two ways could be correlated to the minimum grain size. Therefore, with increasing growth times, the E_g_ is decreased; this is, most probably, due to increasing the grain size and the tensile stress [57]. Generally, the optical E_g_ of a ZnO nanostructure is lower than the ZnO bulk structure, which is about 3.37 eV. This is owing to the quantum confinement effect, which appears once the size of ZnO is reduced to the nanoscale. The E_g_ obtained with the absence of air bubbles is in good agreement with other previous works [58].

### 3.6. Photodetection Characterizations of UV Photodetectors Based on ZnO Nanorods

**Figure 17:**
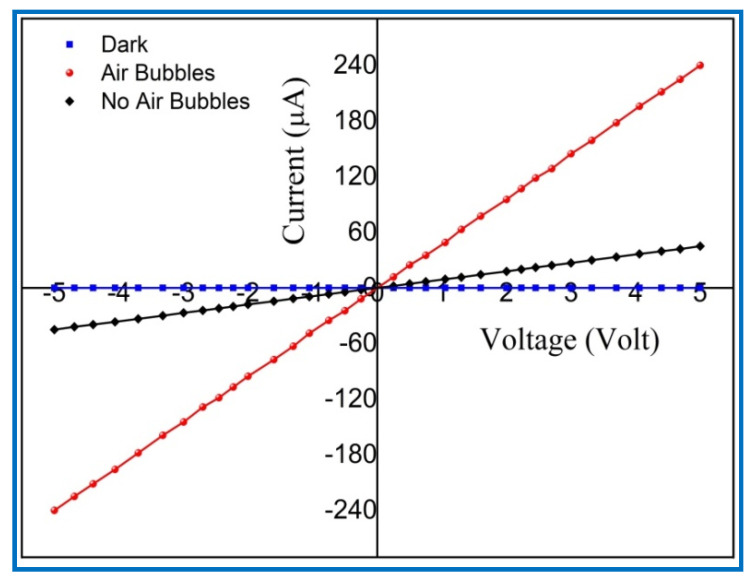
The current-voltage (I-V) characteristics of the (Al/ZnO nanorod) UV photodetectors under dark and UV illumination (380 nm, 0.61 mW/cm^2^).

The Al/ZnO UV PDs were fabricated using ZnO NRs based on the optimum growth duration, and, for both methods, during a CBD process at 95 °C and 4 h of growth time. The metal-semiconductor junction shows the Ohmic behavior in the state of the barrier that is resultant in zero connection. Typically, the Ohmic contact relies upon the type of metal used as a contact and the electron affinity of the semiconductor [59]. In this case, the charge carriers needed to be free to move out from the semiconductor and thus across the contact at the lowest resistance [60]. Because the ZnO is an n-type semiconductor, the work function value of the utilized metal contact needed to be reduced or close to the work function of the semiconductor to obtain the Ohmic behavior. The work function value of Al metal contact is 4.08 eV [61], which is smaller than the value of work function of 4.45 eV for ZnO nanowires, which were described by Ju et al. [62]. Figure 17 shows the linear behavior of current-voltage (I-V) characteristics of the fabricated UV PD devices along with the bias voltage of UV device in the range of −5 to 5 V with no air bubbles involved and with air bubbles involved. This linear behavior of (I-V) characteristics with forward and reverse bias voltage indicates the Ohmic contact between the aluminum (Al) electrodes and the ZnO NRs’ networks. Due to the high electrical sensitivity of metallic semiconductors or the resistance of optical detectors under light through to dark conditions, the Ohmic contact possesses negligible junction resistance [63]. Moreover, the Ohmic contact behavior is essential to encourage the photosensing characteristics of UV photodetectors [64].

At 5 V bias voltage, the measured dark current (I_d_) of fabricated UV PDs with no air bubbles and with air bubbles were 11.254 μA and 7.2831 μA, respectively. It was observed that the minimum dark current is obtained from UV PDs when air bubbles are involved in the growth of ZnO NRs. The minimum (I_d_) is helpful to increase the signal of the sensor to noise (S/N) ratio [65]. Under the UV light illumination with intensity (0.61 mW/cm^2^) and at 5 V bias voltage, the generated photocurrent (I_ph_) of the UV detectors without and with air bubbles was 45.10988 μA and 238.36213 μA, correspondingly. High photocurrent (I_ph_) from UV PDs was obtained when the air bubble was added through ZnO synthesis process. High photocurrent (I_ph_) of the UV device is referred to as high quality and high surface area-to-volume ratio of fabricated ZnO NRs. The responsivity (R) of UV photodetectors was determined using the following equation [66]:

(13) $$R=\frac{I_{\mathrm{ph}}\left(A\right)}{P_{\mathrm{inc}}\left(W\right)}=\frac{I_{\mathrm{ph}\left(A\right)}}{E(W/{\mathrm{cm}}^{2}A\left({\mathrm{cm}}^{2}\right)}$$

where I_ph_ is the photocurrent, P_inc_ is the incident optical power, and A is an active area of the utilized device.

**Figure 18:**
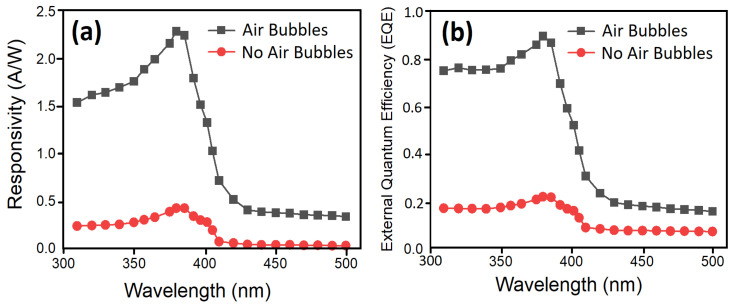
(**a**) Spectral responsivity and (**b**) external quantum efficiency (EQE) of the fabricated UV PDs as a function of incident light source wavelength with no air bubbles and with air bubbles at fixed 5 V bias voltage and under 0.61 mW/cm^2^ UV light illumination.

The light responsivities of fabricated UV sensors as a function of wavelength for two methods at a fixed applied bias voltage of 5 V are shown in Figure 18. Additionally, the external quantum efficiency (EQE), which is the number of free carriers generated by one photon, of the devices was calculated from the measured responsivity (*R*) as EQE = *Rhc/eλ* and plotted against the wavelength of the incident light (Figure 18). Here, *e*, *h*, *λ*, and *c* are electron charge, Planck’s constant, wavelength, and speed of light, respectively [67].

It can be seen from Figure 18 that the EQE in the sample with air bubbles reached values nearly 90% in the wavelength near 382 nm which can be utilized in the UV region. In the sample with no air bubbles, the EQE was close to 20% at 386 nm, which is much lower than the modified sample. It was noted that the responsivity of PDs increases when the wavelength of the incident light source increases. Later, it reached its maximum value at 380 nm, and then it dropped sharply or cutoff at 382 nm and 386 nm for no air bubbles and for air bubbles, correspondingly. The cutoff responsivity near 382 nm and 386 nm are matched with the absorption edge or optical E_g_ of ZnO NRs grown through both methods, respectively. In both methods, the light possesses inadequate energy to stimulate the electron from the valance band to the conduction band, which donates to decrease in the photocurrent of the device at higher wavelengths. The sharp drop in the responsivity spectrum of UV PDs with air bubbles means that the highly photosensitive device is fabricated. The penetration of UV light depth turns out to be narrower and the absorption coefficient increases when the wavelength is decreased, which leads to a growth in the concentration of carrier near the surface of ZnO nanorods [68]. Consequently, a lifetime of photogenerated carriers reduces and causes a drop in responsivity.

**Figure 19:**
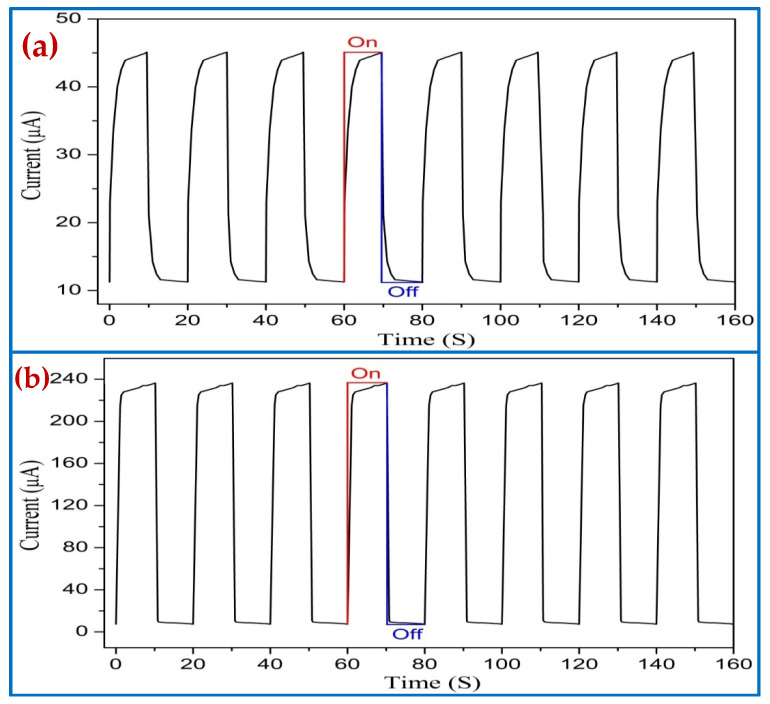
Repeatability properties (on/off) of the Al/ZnO nanorods’ UV PDs under pulsed UV light (380 nm) for optimum growth duration of 4 h: (**a**) no air bubbles and (**b**) air bubbles.

The UV photodetectors devices exhibited a responsivity value of 0.43246 A/W and 2.2851 A/W to 380 nm wavelength at 5 V with no air bubble and with air bubbles, respectively. It was noticed that the responsivity for UV PDs based on ZnO NRs for air bubbles is higher than those reported previously [69,70,71]. The higher value of responsivity (R) is as a result of the high quality of the crystal growth, vertical alignment, uniform density distribution, very similar size, and hexagonal shape of ZnO NRs, which offer high density for big areas and irregular surfaces along with a decent structure of (Al/ZnO NRs) UV photodetectors. The optical reply was investigated by means of dynamic response time measurement under pulsed UV light illumination (0.61 mW/cm^2^ UV light intensity and 380 nm wavelength) to test the excellent stability and reversibility of the UV photodetector under 5 V bias voltage for two ways. Figure 19 demonstrates the corresponding increase photocurrent in accordance with the two ways while the UV light source was switched continually on and off eight times. The duration time between on and off was 10 s. For both cases, it was observed that the UV photodetector has outstanding steadiness and repeatability performance for air bubbles compared with no air bubbles. The results showed that all the curves display acceptable differences with time and photocurrent values, as both cases on/off cycle are stable and repeatable. The rectangular profiles are exhibited for all curves. In all cases, the photocurrent of the UV photodetector was rapidly increased to saturation upon exposure UV light (380 nm) and then exponentially decreased again under dark conditions.

**Figure 20:**
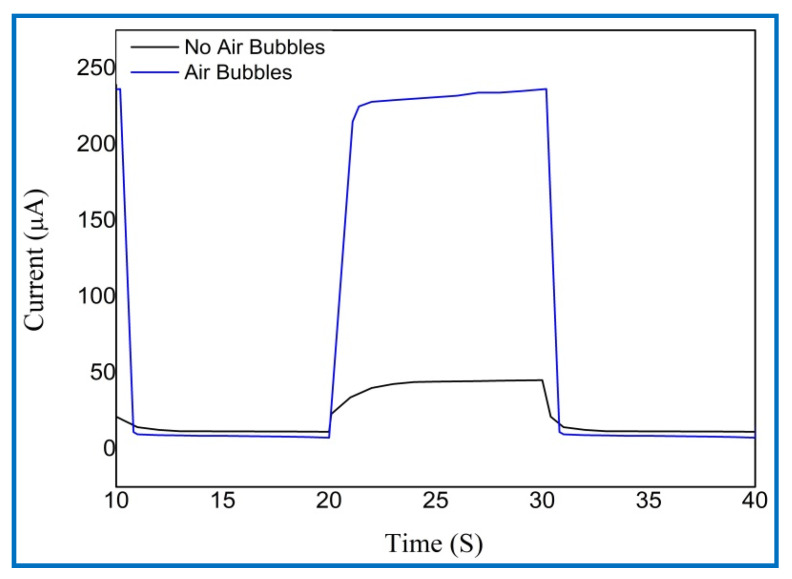
The magnified (on/off) photoresponse cycle for the fabricated UV photodetectors based on ZnO nanorods grown with no air bubbles, with oxygen bubbles, and with air bubbles.

The rise (response) time represents the time of the current to grow from 10% to 90% of its saturation value, while the fall (recovery) time signifies the time for the current to decrease from 90% to 10% of its overload value of UV PDs for the two cases, as shown in Table 3. At a 5 V bias voltage, the UV photodetector grown with air bubbles showed a faster response (rise) and fall (recovery) times of 0.9684 s and 0.5037 s, respectively, compared with that with no air bubbles. One complete photoresponse cycle shows the photocurrent rise and fall edges of UV PDs, as shown in Figure 20.

The sensitivity of the fabricated device can be evaluated by utilizing the following equation [72]:

(14) $$S\left(\%\right)=\frac{I_{\mathrm{ph}}-I_{d}}{I_{d}}\times 100$$

The current gain measured from I-V characteristic is represented by the following equation [73]:

(15) $$\mathrm{Gain}=\frac{I_{\mathrm{ph}}}{I_{d}}$$

The long holes lifetime (τ) and the short electron transit time (τ_t_) may cause high gain (G), a number of electrons perceived per incident photon, along with the following equation [73]:

(16) $$G=\frac{\tau }{\tau_{t}}$$

This gain is straightforwardly related to both quantum efficiency and responsivity as the following equation [73]:

(17) $$R=\eta \left(\frac{q\lambda }{\mathrm{hc}}\right)G$$

In this study, the UV photodetector based on ZnO NRs grown by the modified CBD method had high responsivity (R), high UV light sensitivity, fast response, and short rise and fall times compared with other values that were obtained from the case with no air bubbles as well as from the reported values from the literature on UV PDs based on ZnO NRs using a glass substrate, as shown in Table 3. The rapid photoresponse of UV PDs in the current investigation was related to the high quality, uniform density distribution, perfect hexagonal shape, and great photoactive surface area of ZnO NR arrays in addition to the vertical well-alignment of ZnO array, which was helpful in the high-speed photoresponse. The high aspect ratio was a result of the high surface-to-volume ratio of ZnO NRs. Therefore, the increased surface area of ZnO NRs leads to the assortment of additional ultraviolet light, followed by growing photocurrent [74]. Additionally, the large surface area-to-volume ratio of ZnO NRs boosts the electron-hole pairs recombination, which donates to a quicker decay time [75]. The high superiority of ZnO NRs results in a decrease to the density of charge trap centers, which is a part of impurities in the crystal structure; thus the photoresponsivity is significantly improved when air bubbles were introduced to the synthesizing process. The impurity in the nanorods’ structure had a strong negative impact on the photoresponse; moreover, the photocurrent, quantum efficiency, and photosensitivity of the fabricated UV PDs based on ZnO NRs for the two cases and for some previous works are summarized in Table 3. From Table 3, it can be noticed that the entire parameters of PDs can clarify the significant improvement of the photodetection appearances of the UV PDs synthesized by the modified CBD method. These results indicated that the Al/ZnO NRs for UV PDs with air bubbles exhibit potential application for the UV photodetectors.

**Table 3:** Comparison between the current study and the literature of the responsivity (R), response time, recovery time, sensitivity (S), and gain for the (Al/ZnO nanorod) UV photodetectors based on ZnO nanorods fabricated by two ways of preparation.

Bias Voltage (V)	λ (nm)	R (A/W)	Response Time (S)	Recovery Time (S)	S %	Gain	Ref.
5	380	0.43246	2.649	1.321	300.834	4.008	No Air Bubbles
**5**	**380**	**2.2851**	**0.9684**	**0.5037**	**3172.811**	**32.728**	**Air Bubbles**
5	365	1.22	-	-	81.26	1.8	[76]
1.8	365	0.199	-	-	109.415	2.095	[77]
5	380	102	55.5	33.1	1720	-	[78]
3	365		41.3	30.7	465.2	-	[79]
2	365	0.22	3	4	-	-	[80]
3	365	-	80	20	-	-	[81]
1	325	1.738	0.032	0.041	20		[82]
5	365	0.0706	-	-	815.4	9.15	[83]
3	325	2.856	1.2	1.8	1175	12.75	[84]
5	365	0.39	3.9	2.6	2319	24.2	[85]
5	365		12	9	1575	-	[86]

## 4. Conclusions

High-quality UV photodetectors based on ZnO NRs have been fabricated using both M-CBD and traditional CBD methods. The ZnO NRs using M-CBD for different growth durations showed significantly improved results over the traditional CBD method under the same growth conditions. This is explained according to the agitation of all deposition solutions, which leads to more homogeneity of the deposition solution during the CBD growth process. In this study, Al/ZnO UV PDs based on ZnO NRs were fabricated with optimal growth duration for both preparation methods. The highest aspect ratio was obtained at 4 h of growth duration, and the aspect ratio of ZnO NRs prepared by M-CBD was higher than the aspect ratio of ZnO NRs grown by the traditional CBD method. Moreover, the results of different growth times showed that the M-CBD method offers higher crystal properties and growth rates with the same duration compared to the traditional method. Explicitly, with one-hour growth time the growth rate for the modified method was 26.2 nm/min, while the growth rate using the traditional method was 8.5 nm/min. Additionally, at 5 V bias voltage the UV PDs based on ZnO NRs fabricated when air bubbles were involved showed high sensitivity, short response time, quick recovery time, high gain, low dark current, high photocurrent, and higher responsivity compared with devices fabricated by the traditional way.

## Figures and Tables

**Table 1:** Lattice Parameters and Structure Properties of ZnO Nanorods of Diffraction Peak (002) for Different Growth Durations where Air Bubbles Did Not Exist.

Growth Duration (h)	2θ	I (a. u)	a (Å)	c (Å)	Ƹ*_a_*%	Ƹ*_c_*%	d (Å)
**1**	34.375	104	3.0100	5.213	−7.483	−0.029	2.606
**2**	34.425	202	3.0058	5.206	−7.625	−0.069	2.603
**3**	34.425	399	3.0058	5.206	−7.625	−0.069	2.603
**4**	34.425	465	3.0058	5.206	−7.625	−0.069	2.603
**5**	34.375	495	3.0100	5.213	−7.483	−0.029	2.606

**Table 2:** Lattice parameters and structure properties of ZnO nanorods of diffraction peak (002) for different growth times where air bubbles existed.

Growth Duration (h)	2θ	I (a. u)	a (Å)	c (Å)	Ƹ*_a_*%	Ƹ*_c_*%	d (Å)
**0.5**	34.375	282	3.01004	5.2135	−7.483	−0.0299	2.6067
**1**	34.382	328	3.00941	5.2125	−7.502	−0.0507	2.6062
**2**	34.383	1373	3.00931	5.2123	−7.505	−0.0541	2.6061
**3**	34.393	2284	3.00853	5.2109	−7.529	−0.0800	2.6055
**4**	34.400	3206	3.00786	5.2097	−7.550	−0.1023	2.6049
**5**	34.425	18,114	3.00580	5.2062	−7.613	−0.1707	2.6031
